# Human Consciousness and Behavior towards Infectious Diseases and Vaccines 2.0: A Commentary from Special Issue Editors

**DOI:** 10.3390/vaccines10030471

**Published:** 2022-03-18

**Authors:** Yutaka Ueda

**Affiliations:** Department of Obstetrics and Gynecology, Graduate School of Medicine, Osaka University, Osaka 565-0871, Japan; y.ueda@gyne.med.osaka-u.ac.jp

The WHO has identified vaccine hesitancy as one of the 10 threats to global health. One of the biggest causes of vaccine hesitancy is an inaccurate understanding of infectious diseases and vaccines. Currently, a new strain of coronavirus has spread around the world. People have a great fear of new coronavirus infections. A protective vaccine against coronavirus would be widely well received, even if there are inevitable severe or unknown adverse reactions. Awareness of various kinds of infectious diseases, preventive behavior against infectious diseases including vaccination, and various kinds of bias towards the recognition of infectious diseases and vaccines might be a potential focus.

Dr. Foteini Malli et al. [1] presented the data from a nationwide observational study, suggesting the beneficial impact of the national vaccination campaign in Greece, which may offer control of the SARS-CoV-2 pandemic. 

Dr. Adam Palanica et al. [2] examined the demographic characteristics, psychological perceptions, and vaccination-related opinions and experiences of a large Canadian sample who had received two initial doses of any COVID-19 vaccine combination and discussed the real-world implications of the results.

Dr. Abanoub Riad et al. [3] conducted machine learning analysis and suggested five important predictors of COVID-19 vaccination willingness among dental students globally, i.e., the economic level of the country where the student lives and studies, the individual’s trust of the pharmaceutical industry, the individual’s misconception of natural immunity, the individual’s belief in vaccines’ risk-benefit-ratio, and the individual’s attitudes toward novel vaccines.

Dr. Georgios Marinos et al. [4] conducted a cross-sectional, questionnaire-based, online study, which was conducted among the members of the Athens Medical Association. The finding that participants reported high reliability of the information related to COVID-19 vaccination provided by the Greek public health authorities is an opportunity which should be broadly exploited by policymakers in order to combat vaccination hesitancy, and further improve COVID-19 vaccination uptake and coverage among physicians/health care workers and the general population.

Dr. Elham Kateeb et al. [5] assessed the predictors related to the willingness of Palestinian dental students to receive the COVID-19 vaccine when it became available, and concluded that adequate information about vaccines, their risk–benefit ratios, and natural and acquired immunity is important to build trust and favorable attitudes towards vaccines among future dentists.

Dr. Abanoub Riad et al. [6] investigated the COVID-19 vaccine hesitancy drivers among university students in the Czech Republic and suggested a fair probability of achieving community immunity (herd immunity) among the target population group. The primary prevention strategies in the Czech Republic need to be culturally sensitive and inclusive of foreign nationals.

Dr. Yen-Ju Lin et al. [7] compared the differences in motivation to receive a COVID-19 vaccination between frontline physicians and nurses and the Taiwanese public. The factors related to the motivation to receive a COVID-19 vaccination should be considered when designing programs to increase motivation to receive a COVID-19 vaccination among frontline health workers and the public.

The editor believes that these findings will provide a rationale for more effective dissemination of vaccines against COVID-19 and hopes that this Special Issue “Human Consciousness and Behavior towards Infectious Diseases and Vaccines 2.0” will contribute to the improvement of health of people around the world.
